# Supplementary material for: Enzymatic Adaptation of Bifidobacterium bifidum to Host Glycans, Viewed from Glycoside Hydrolyases and Carbohydrate-Binding Modules
Source: Microorganisms. 2020 Mar 28;8(4):481. doi: 10.3390/microorganisms8040481 (PMC7232152; doi:10.3390/microorganisms8040481)
Supplement: Supplementary file 1 [file microorganisms-08-00481-s001.pdf]

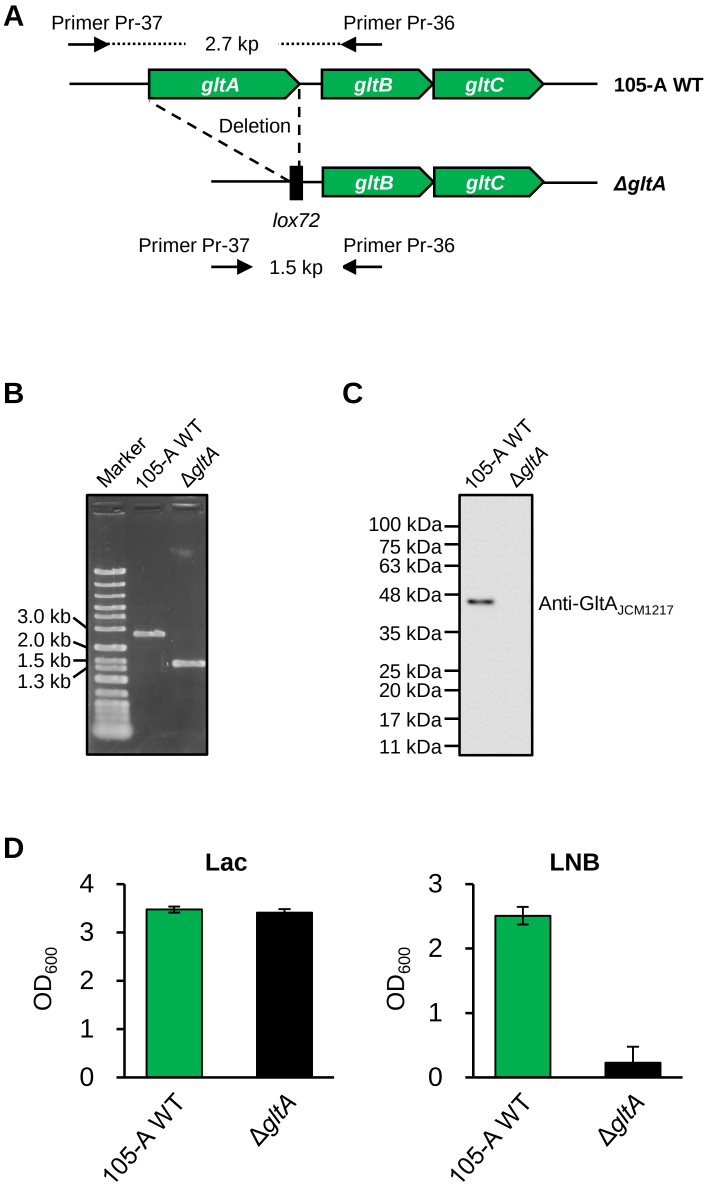

Supplementary Figure S1. Disruption of *gltA* in *B. longum* 105-A. (a) Schematic drawing of the *gltABC* loci on the chromosomes of *B. longum* 105-A wild-type and Δ*gltA* strains. (b) Disruption of *gltA* was verified by the genomic PCR followed by agarose gel electrophoresis. The primer pairs Pr-36 and Pr-37 were used to amplify the *gltA* region. (c) Western blot analysis using anti-GltA antibodies [89]. The cell-free extracts (1.6 μg) of the wild-type and Δ*gltA* strains were loaded onto SDS-polyacrylamide gel electrophoresis. (d) Growth of the wild-type and Δ*gltA* strains in a basal media supplemented with 1 % Lac or LNB. OD_{600} values after 60 h incubation are shown. Data are mean ± SD of biological triplicates.
